# Vector critical points and generalized quasi-efficient solutions in nonsmooth multi-objective programming

**DOI:** 10.1186/s13660-017-1456-2

**Published:** 2017-08-09

**Authors:** Zhen Wang, Ru Li, Guolin Yu

**Affiliations:** Institute of Applied Mathematics, North Minzu University, Yinchuan, Ningxia 750021 P.R. China

**Keywords:** 90C29, 90C46, 26B25, vector variational-like inequality, multi-objective programming, approximate invexity, quasi-efficiency, vector critical point

## Abstract

In this work, several extended approximately invex vector-valued functions of higher order involving a generalized Jacobian are introduced, and some examples are presented to illustrate their existences. The notions of higher-order (weak) quasi-efficiency with respect to a function are proposed for a multi-objective programming. Under the introduced generalization of higher-order approximate invexities assumptions, we prove that the solutions of generalized vector variational-like inequalities in terms of the generalized Jacobian are the generalized quasi-efficient solutions of nonsmooth multi-objective programming problems. Moreover, the equivalent conditions are presented, namely, a vector critical point is a weakly quasi-efficient solution of higher order with respect to a function.

## Introduction

Convexity and its generalizations played a critical role in multi-objective programming problems. In many generalizations, approximate convexity and invexity are two significant generalized versions of convexity, which tried to weaken the convexity hypotheses thus to study the relations between vector variational-like inequalities and multi-objective programming problems. Invexity was firstly put forward by Hanson [1]. Then Osuna-Gómez *et al.* [2] introduced the notions of generalized invexity for differentiable functions in a finite-dimensional contex. And this generalized invexity has been extended to locally Lipschitz functions using the generalized Jacobian (see [3, 4]). Ben-Israel and Mond [5] presented pseudoinvex functions which generalized pseudoconvex functions in the same manner as invex functions generalized convex functions. Mishra *et al.* [6] and Ngai *et al.* [7] introduced the concept of approximately convex functions. Inspired and motivated by this ongoing research work, we present the concept of approximately invex function of higher order.

The notion of an efficient solution in multi-objective programming is widely used. Considering the complexity of optimization problems, several variants of the efficient solutions have been introduced (see [8–11]). Recently, researchers have shown great interests in quasi-efficiency of multi-objective programming (see [12, 13]). In this work, we give the notion of a quasi-efficient solution of higher order for a class of *nonsmooth multi-objective programming problems* (NMPs) with respect to a function.

The vector variational inequality was initially introduced by Giannessi [14]. Since then vector variational inequalities, which were used as an efficient tool to study multi-objective programming, have attracted much attention and have been extended to *generalized vector variational-like inequalities* (GVVI). Recently, a great quantity of work focused on the study of relations between (GVVI) and multi-objective programming under different convexity assumptions (see [15–17]). Motivated by the previous contributions, in this note, our purpose is to obtain the relations between (GVVI) and (NMP) under approximate invexity of higher order.

The rest of this work is organized as follows. In Section 2, we recall some basic definitions and preliminary results. Besides, the notions of approximately invex function of higher order with respect to vector-valued functions and (weakly) quasi-efficient solution of higher order for (NMP) with respect to a vector-valued function are introduced, and examples are provided to illustrate their existence. In Section 3, the relations between (GVVI) and (NMP) are established under the approximate invexity of higher-order assumptions. In Section 4, we study the relations between vector critical points and weakly quasi-efficient solutions of higher order for (NMP) with respect to a vector-valued function.

## Notations and preliminaries

Throughout the current paper, unless otherwise stated, $\mathbb{R}$, $\mathbb{R}^{n}$, $\mathbb{R}^{n}_{+}$ stand for the set of all real numbers, the *n*-dimensional Euclidean space and the nonnegative orthant of $\mathbb{R}^{n}$, respectively. For any $x, y\in\mathbb {R}^{n}$, the inner product of *x* and *y* is denoted $x^{T}y$, where the superscript *T* represents the transpose of a vector. Let $X\subseteq \mathbb{R}^{n}$ be a nonempty subset and $m\geq1$ be a positive integer, the symbols $\operatorname{co}(X)$ and $\operatorname{int}(X)$ represent the convex hull of *X* and the interior of *X*, respectively. We employ the following conventions for vectors in $\mathbb{R}^{n}$:

$$\begin{aligned}& x= y \quad \Leftrightarrow\quad x_{i}= y_{i}, \quad \forall i= 1,\ldots,n; \\& x\leqq y \quad \Leftrightarrow\quad x_{i}\leq y_{i}, \quad \forall i=1,\ldots,n; \\& x\leqslant y\quad \Leftrightarrow \quad x_{i}\leq y_{i}, \quad \forall i=1,\ldots,n, i\neq j \mbox{ and }x_{j}< y_{j} \mbox{ for some } j; \\& x< y \quad \Leftrightarrow \quad x_{i}< y_{i}, \quad \forall i= 1,\ldots,n. \end{aligned}$$

For the sake of convenience, we firstly recall some notations that will be used in the sequel. *We always suppose that* $f: X\rightarrow \mathbb{R}^{p}$ *,* $\eta: X\times X\rightarrow\mathbb{R}^{n}$ *and* $\psi: X\times X\rightarrow\mathbb{R}^{n}$ *are vector-valued functions in the rest of this paper*.

### Definition 2.1

see [18]

The function $f: X\rightarrow\mathbb{R}^{p}$ is said to be locally Lipschitz on *X*, if for every $x\in X$ there exist a neighborhood $U_{x}\subseteq X$ of *x* and a constant $L>0$ such that, for all *y*, $z\in U_{x}$,

2.1 $$ \bigl\Vert f(y)-f(z) \bigr\Vert \leq L \Vert y-z \Vert . $$

Rademacher’s theorem (see Corollary 4.12 in [19]) indicates that a function *f* satisfying the Lipschitz condition (2.1) is Fréchet differentiable. Based on this fact, Clarke [18] presented the following concept of the generalized Jacobian of *f* at some point.

### Definition 2.2

see [18]

Let $x_{0}\in X$ and $Jf(x)$ represent the usual Jacobian matrix of *f* at *x* whenever *f* is Fréchet differentiable at *x*. The generalized Jacobian of *f* at $x_{0}$ is

$$ \partial_{J} f(x_{0})=\operatorname{co} \Bigl\{ \lim _{n\rightarrow\infty}Jf(x_{n}): x_{n}\rightarrow x_{0}, Jf(x_{n})\mbox{ exists}\Bigr\} . $$

Let a scalar function $\varphi: X\rightarrow\mathbb{R}$ be locally Lipschitz at $x_{0}$, then the upper Clarke directional derivative of *φ* at $x_{0}$ in the direction $v\in\mathbb{R}^{n}$ is given by

$$ \varphi'(x_{0},v)=\lim_{x\rightarrow x_{0}}\sup _{t\downarrow0}\frac {\varphi(x+tv)-\varphi(x)}{t}, $$

and the Clarke subdifferential of *φ* at $x_{0}$, denoted by $\partial\varphi(x_{0})$, is defined as follows:

$$ \partial\varphi(x_{0})=\bigl\{ \xi\in\mathbb{R}^{n}: \varphi'(x_{0},v)\geq \langle\xi,v\rangle, \forall v\in \mathbb{R}^{n}\bigr\} . $$

For a vector-valued function $f=(f_{1},\ldots,f_{p})^{T}: X\rightarrow\mathbb {R}^{p}$, its Clarke subdifferential is the cartesian product of Clarke subdifferentials of the components $f_{i}: X\rightarrow\mathbb{R}$, $i=1,2,\ldots,p$ of *f*, that is, $\partial f(x)=\partial f_{1}(x)\times \cdots\times\partial_{p}f(x)$. It has been shown in [18] that, for a scalar function $\varphi: X\rightarrow\mathbb{R}$, $\partial _{J}\varphi(x)=\partial\varphi(x)$, but for the vector-valued function *f*, $\partial_{J} f(x)$ is contained and is different from $\partial f(x)$.

### Definition 2.3

see [20]

The subset $\emptyset\neq X\subseteq \mathbb{R}^{n}$ is said to be invex with respect to $\eta: X\times X\rightarrow\mathbb{R}^{n}$, if for every $x, y\in X$, $\lambda\in[0, 1]$, we have

$$ y+\lambda\eta(x, y)\in X. $$

*From now on, we always assume that the subset* $X\subseteq\mathbb {R}^{n}$ *is a nonempty invex set with respect to some* *η* *unless otherwise specified.*

The generalized invexity of differentiable functions in a finite-dimensional space (see [2]) has been extended to locally Lipschitz functions using the generalized Jacobian as follows (see [3, 4]).

### Definition 2.4

see [4]

Let $x_{0}\in X$ and $\eta: X\times X\rightarrow\mathbb{R}^{n}$. The function $f: X\rightarrow\mathbb{R}^{p}$ is said to be invex at $x_{0}$ with respect to *η*, if for all $x\in X$,

$$ f(x)\geqq f(x_{0})+A\eta(x,x_{0}),\quad \forall A\in \partial_{J} f(x_{0}). $$

*f* is said to be invex with respect to *η* on *X*, if for every $x\in X$, *f* is invex at *x* with respect to *η*.

### Definition 2.5

see [4]

Let $x_{0}\in X$ and $\eta: X\times X\rightarrow\mathbb{R}^{n}$. The function $f: X\rightarrow\mathbb{R}^{p}$ is said to be pseudoinvex at $x_{0}$ with respect to *η*, if for all $x\in X$,

$$ A\eta(x,x_{0})\geqq0,\quad \mbox{for some } A\in\partial_{J} f(x_{0})\quad \Rightarrow\quad f(x)\geqq f(x_{0}), $$

or equivalently,

$$ f(x)< f(x_{0})\quad \Rightarrow \quad A\eta(x,x_{0})< 0, \quad \forall A\in\partial_{J} f(x_{0}). $$

*f* is said to be pseudoinvex with respect to *η* on *X*, if for every $x\in X$, *f* is pseudoinvex at *x* with respect to *η*.

In the generalized convexity of functions, the study of approximately convex functions (see [6, 7, 12, 21]) is a hot spot. Mishra and Upadhyay [21] introduced the following concept of vector-valued approximately convex functions.

The function $f:X\rightarrow\mathbb{R}^{p}$ is said to be approximately convex at $x_{0}\in X$, if for all $\alpha\in \operatorname{int}(\mathbb{R}^{p}_{+})$ such that

$$ f(x)\geqq f(x_{0})+\xi^{T}(x-x_{0})-\alpha \|x-x_{0}\|, \quad \forall x\in X , \forall\xi\in\partial f(x_{0}). $$

Motivated by above definitions, we give the notions of approximate invexity of order *m* with respect to *η* and *ψ*, strictly approximate invexity of order *m* with respect to *η* and *ψ* and approximate pseudoinvexity of type I of order *m* with respect to *η* and *ψ* as follows.

### Definition 2.6

Let $x_{0}\in X$ and $m\geq1$ be a positive integer. The function $f: X\rightarrow\mathbb{R}^{p}$ is said to be approximately invex of order *m* at $x_{0}$ with respect to *η* and *ψ*, if there exist $\eta: X\times X\rightarrow\mathbb{R}^{n}$, $\psi: X\times X\rightarrow\mathbb {R}^{n}$ and $\alpha\in \operatorname{int}(\mathbb{R}^{p}_{+})$ such that, for all $x\in X $,

$$ f(x)\geqq f(x_{0})+A\eta(x,x_{0})-\alpha \bigl\Vert \psi(x,x_{0}) \bigr\Vert ^{m}, \quad \forall A\in \partial_{J} f(x_{0}). $$

*f* is said to be approximately invex of order *m* with respect to *η* and *ψ* on *X*, if for every $x\in X$, *f* is approximately invex of order *m* at *x* with respect to *η* and *ψ*.

### Remark 2.1

Replacing $A\in\partial_{J} f(x_{0})$ by $\xi\in\partial f(x_{0})$, setting $\eta(x,x_{0})=x-x_{0}$, $\psi(x,x_{0})=x-x_{0}$ and $m=1$ in Definition 2.6, then we arrive at the notion of approximately convex function, defined by Mishra and Upadhyay [21].

### Remark 2.2

A function which is invex at $x_{0}$ with respect to *η* is also approximately invex of order *m* at $x_{0}$ with respect to *η* and *ψ*, but in the contrary case, it does not hold. The following example is given to illustrate this fact.

### Example 2.1

Consider the vector-valued function $f:\mathbb{R}\rightarrow\mathbb {R}^{2}$, defined by $f(x)=(x,\varphi(x))^{T}$, where

$$ \varphi(x)= \textstyle\begin{cases} -x& \mbox{if } x< 0, \\ 0& \mbox{if }0\leq x \leq1, \\ 2-x& \mbox{if }x>1. \end{cases} $$

It can easily be seen that

$$ \partial_{J} f(x)= \textstyle\begin{cases} \{(1, -1)^{T}\}& \mbox{if } x< 0 \mbox{ or } x>1, \\ \{(1, a)^{T}: -1\leq a\leq0\}& \mbox{if } x=0 \mbox{ or } x=1, \\ \{(1, 0)^{T}\}& \mbox{if } 0< x< 1. \end{cases} $$

We firstly prove that *f* is not invex with respect to $\eta:\mathbb {R}\times\mathbb{R}\rightarrow\mathbb{R}$ on $\mathbb{R}$. Indeed, choose $\bar{x}=0$ and $x_{0}=1$, then there exists no $u=\eta(\bar {x},x_{0})\in\mathbb{R}$ such that $f(\bar{x})-f(x_{0})\geqq Au$ for every $A=(1, a)^{T}\in\partial_{J} f(x_{0})$. In other words, if $(-1,0)^{T}\geqq (u,au)^{T}, \forall a\in[-1,0]$, this implies $-1\geqq u$ and $0\geqq au$. For $a=-1$, from above inequality $0\geqq au$ we obtain $u\geqq 0$, which contradicts $-1\geqq u$. Finally, we show that *f* is approximately invex of order *m* at $x_{0}=1$ with respect to $\eta:\mathbb{R}\times\mathbb{R}\rightarrow \mathbb{R}$ and $\psi:\mathbb{R}\times\mathbb{R}\rightarrow\mathbb{R}$. Take $\eta(x,x_{0})=1$, $\psi(x,x_{0})=x-x_{0}-1=x-2$, $m=1$ and $\alpha =(\alpha_{1},\alpha_{2})^{T}=(1,2)^{T}$. From the above we have $A=(1, a)^{T}\in\partial_{J} f(1)$, $-1\leq a\leq0$. then we can see the following inequality holds true invariably:

$$\begin{aligned} f(x)-f(x_{0})-A\eta = & \bigl(x-2,\varphi(x)-a\bigr)^{T} \\ \geqq & \bigl(- \vert x-2 \vert ,-2|x-2|\bigr)^{T} \\ =& -\alpha \bigl\Vert \psi(x,x_{0}) \bigr\Vert . \end{aligned}$$

### Definition 2.7

Let $x_{0}\in X$ and $m\geq1$ be a positive integer. The function $f: X\rightarrow\mathbb{R}^{p}$ is said to be strictly approximately invex of order *m* at $x_{0}$ with respect to *η* and *ψ*, if there exist $\eta: X\times X\rightarrow\mathbb{R}^{n}$, $\psi: X\times X\rightarrow \mathbb{R}^{n}$ and $\alpha\in \operatorname{int}(\mathbb{R}^{p}_{+})$ such that, for all $x\in X $,

$$ f(x)> f(x_{0})+A\eta(x,x_{0})-\alpha \bigl\Vert \psi(x,x_{0}) \bigr\Vert ^{m}, \quad x\neq x_{0}, \forall A\in\partial_{J} f(x_{0}). $$

*f* is said to be strictly approximately invex of order *m* with respect to *η* and *ψ* on *X*, if for every $x\in X$, *f* is strictly approximately invex of order *m* at *x* with respect to *η* and *ψ*.

### Definition 2.8

Let $x_{0}\in X$ and $m\geq1$ be a positive integer. The function $f: X\rightarrow\mathbb{R}^{p}$ is said to be approximately pseudoinvex type I of order *m* at $x_{0}$ with respect to *η* and *ψ*, if there exist $\eta: X\times X\rightarrow\mathbb{R}^{n}$, $\psi: X\times X\rightarrow\mathbb{R}^{n}$ and $\alpha\in \operatorname{int}(\mathbb{R}^{p}_{+})$ such that, for all $x\in X $,

$$ A\eta(x,x_{0})\geqq0, \quad \mbox{for some } A\in \partial_{J} f(x_{0})\quad \Rightarrow\quad f(x)-f(x_{0})\geqq-\alpha \bigl\Vert \psi(x,x_{0}) \bigr\Vert ^{m}, $$

or, equivalently,

$$ f(x)+\alpha \bigl\Vert \psi(x,x_{0}) \bigr\Vert ^{m}< f(x_{0})\quad \Rightarrow\quad A\eta(x,x_{0})< 0, \quad \forall A\in\partial_{J} f(x_{0}). $$

*f* is said to be approximately pseudoinvex type I of order *m* with respect to *η* and *ψ* on *X*, if for every $x\in X$, *f* is approximately pseudoinvex type I of order *m* at *x* with respect to *η* and *ψ*.

The following example illustrates the existence of approximate invexity of order *m* with respect to *η* and *ψ* and of an approximately pseudoinvex type I function of order *m* with respect to *η* and *ψ*.

### Example 2.2

Let $X=\mathbb{R}$, $\alpha=(\alpha_{1},\alpha_{2})^{T}>0$ and $m\geq1$ be a positive integer. Consider the following functions: $f: X\rightarrow\mathbb{R}^{2}$, $f': X\rightarrow\mathbb{R}^{2}$, $\eta: X\times X\rightarrow\mathbb{R}$ and $\psi: X\times X\rightarrow \mathbb{R}^{2}$ defined by

$$ \begin{aligned} &f(x)=\bigl(x, \max\{-x,0,x-1\}\bigr)^{T},\qquad f'(x)= \bigl(-2x, \max\{-x,0,x-1\}\bigr)^{T}, \\ &\eta(x, x_{0})=x-x_{0} \quad \mbox{and} \quad \psi(x,x_{0})=\biggl(\frac{x^{2}}{\sqrt{1+x_{0}^{2}}},0\biggr)^{T}. \end{aligned} $$

For any positive integer $m\geq1$, it is easy to verify that *f* is approximately invex of order *m* at $x_{0}=-1$ with respect to *η* and *ψ*. In fact, we can easily obtain $A=(1, -1)^{T}\in\partial _{J}f(x_{0})$. By direct calculation, we derive

$$\begin{aligned}& f(x)-f(x_{0})=\bigl(x, \max\{-x,0,x-1\}\bigr)^{T}-(-1,1)^{T}= \bigl(x+1, \max\{-x,0,x-1\} -1\bigr)^{T}, \\& A\eta(x,x_{0})-\alpha \bigl\Vert \psi(x,x_{0}) \bigr\Vert ^{m} = (1,-1)^{T}(x+1)-(\alpha_{1}, \alpha_{2})^{T} \biggl(\biggl(\frac{x^{2}}{\sqrt{2}} \biggr)^{2}+0^{2} \biggr)^{\frac {m}{2}} \\& \hphantom{A\eta(x,x_{0})-\alpha \bigl\Vert \psi(x,x_{0}) \bigr\Vert ^{m}} = \bigl(x+1, -(x+1)\bigr)^{T}-(\alpha_{1}, \alpha_{2})^{T}\biggl(\frac{x^{2}}{\sqrt {2}}\biggr)^{m}. \end{aligned}$$

Obviously,

$$ \bigl(x+1, \max\{-x,0,x-1\}-1\bigr)^{T}\geqq\bigl(x+1, -(x+1) \bigr)^{T}. $$

Furthermore, because of $\alpha=(\alpha_{1},\alpha_{2})^{T}>0$, we can arrive at

$$ \bigl(x+1, \max\{-x,0,x-1\}-1\bigr)^{T}\geqq\bigl(x+1, -(x+1) \bigr)^{T}-(\alpha_{1}, \alpha _{2})^{T} \biggl(\frac{x^{2}}{\sqrt{2}}\biggr)^{m}. $$

That is,

$$ f(x)\geqq f(x_{0})+A\eta(x,x_{0})-\alpha \bigl\Vert \psi(x,x_{0}) \bigr\Vert ^{m}, \quad \forall x\in X, \forall A\in\partial_{J} f(x_{0}). $$

So, we have verified that *f* is approximatelyly invex of order *m* at $x_{0}=-1$ with respect to *η* and *ψ*.

Now, let us prove that $f'(x)=(-2x, \max\{-x,0,x-1\})^{T}$ is approximately pseudoinvex type I of order *m* at $x_{0}=0$ with respect to $\eta(x, x_{0})=x-x_{0}$ and $\psi(x,x_{0})=(\frac{x^{2}}{\sqrt {1+x_{0}^{2}}},0)^{T}$. Actually, it is not difficult to get $A=(-2, a)^{T}\in\partial_{J}f'(x_{0})=\{(-2, a)^{T}:-1\leq a\leq0\}$. We suppose that

$$ A\eta(x,x_{0})=(-2, a)^{T}x=(-2x, ax)^{T} \geqq0, $$

then we arrive at

$$\begin{aligned} f'(x)-f'(x_{0}) =&\bigl(-2x, \max\{-x,0,x-1 \}\bigr)^{T} \geqq-(\alpha_{1}, \alpha_{2})^{T} \biggl(\frac{x^{2}}{\sqrt{2}}\biggr)^{m} \\ = &-\alpha \bigl\Vert \psi(x,x_{0}) \bigr\Vert ^{m}. \end{aligned}$$

This fulfills the condition of an approximately pseudoinvex type I of order *m* function at $x_{0}=0$ with respect to *η* and *ψ*.

### Remark 2.3

It is obvious that if $f:X \rightarrow\mathbb{R}^{p}$ is pseudoinvex at $x_{0}\in X$ with respect to *η*, then it is also approximately pseudoinvex type I of order *m* at $x_{0}$ with respect to *η* and *ψ*. But the converse does not hold. For example, consider $\varphi :X\rightarrow\mathbb{R}$, given by

$$ \varphi(x)= \textstyle\begin{cases} -x^{2}& \mbox{if } x< 0, \\ \sin x& \mbox{if } 0\leq x \leq2\pi. \end{cases} $$

Taking $\eta(x,x_{0})=x-x_{0}$, $\psi(x,x_{0})=x-x_{0}$ and $m=1$, then *φ* is approximately pseudoinvex type I of order *m* at $x_{0}=0$ with respect to *η* and *ψ*. As for any $\alpha>0$, there exists $\delta=\min(\pi,\alpha)>0$ such that $A\eta(x,x_{0})\geq0$ for some $A\in \partial_{J}\varphi(x_{0})$ implies

$$ \varphi(x)-\varphi(x_{0})\geq-\alpha\|x-x_{0}\|, \quad \forall x \in B(x_{0},\delta)\cap X. $$

However, *φ* is not pseudoinvex at $x_{0}$ with respect to *η*. Indeed, for every $\delta>0$, there exists $x\in B(x_{0},\delta)\cap X$ such that $A\eta(x,x_{0})\geq0$, $A\in\partial_{J}\varphi(x_{0})$ does not imply $\varphi(x)\geq\varphi(x_{0})$ (see Remark 3 in [22]).

We consider the following *nonsmooth multi-objective programming problem* (NMP):

$$ (\mathrm{NMP}) \quad \textstyle\begin{cases} \mathcal{}\mbox{minimize } f(x)=(f_{1}(x),f_{2}(x),\ldots ,f_{p}(x))^{T}, \\ \quad \mbox{subject to } x\in X, \end{cases} $$

where $f_{i}:X \rightarrow\mathbb{R}$, $i\in P=\{1,2,\ldots,p\}$ are non-differentiable functions.

In multi-objective programming problems, efficient and weakly efficient solutions are widely used. Considering the complexity of the optimization problem in reality and in order to find the optimal solution of multi-objective optimization problem in a smaller range, the notion of quasi-efficient and weakly quasi-efficient are introduced as follows (see [12, 21, 22]).

### Definition 2.9

A point $x_{0}\in X$ is said to be an efficient solution to the (NMP), if there exists no $x\in X$ such that

$$ f(x)\leqslant f(x_{0}). $$

### Definition 2.10

A point $x_{0}\in X$ is said to be a weakly efficient solution to the (NMP), if there exists no $x\in X$ such that

$$ f(x)< f(x_{0}). $$

### Definition 2.11

Let $x_{0}\in X$.

(i) A point $x_{0}$ is said to be a quasi-efficient solution to the (NMP), if there exists $\alpha\in \operatorname{int}(\mathbb{R}^{p}_{+})$ such that, for any $x\in X$, the following cannot hold:
  $$ f(x)\leqslant f(x_{0})-\alpha\|x-x_{0}\|. $$
(ii) A point $x_{0}$ is said to be a weakly quasi-efficient solution to the (NMP), if there exists $\alpha\in \operatorname{int}(\mathbb{R}^{p}_{+})$ such that, for any $x\in X$, the following cannot hold:
  $$ f(x)< f(x_{0})-\alpha\|x-x_{0}\|. $$

Now, we present the concepts of (weakly) quasi-efficient solution of order *m* with respect to a function *ψ* for the problem (NMP).

### Definition 2.12

Let $m\geq1$ be a positive integer. A point $x_{0}\in X$ is called a quasi-efficient solution of order *m* for (NMP) with respect to *ψ*, if there exist a function $\psi: X\times X\rightarrow\mathbb{R}^{n}$ and $\alpha\in \operatorname{int}(\mathbb{R}^{p}_{+})$ such that, for any $x\in X$, the following cannot hold:

$$ f(x)\leqslant f(x_{0})-\alpha \bigl\Vert \psi(x,x_{0}) \bigr\Vert ^{m}. $$

### Definition 2.13

Let $m\geq1$ be a positive integer. A point $x_{0}\in X$ is called a weakly quasi-efficient solution of order *m* for (NMP) with respect to *ψ*, if there exist a function $\psi: X\times X\rightarrow\mathbb {R}^{n}$ and $\alpha\in \operatorname{int}(\mathbb{R}^{p}_{+})$ such that, for any $x\in X$, the following cannot hold:

$$ f(x)< f(x_{0})-\alpha \bigl\Vert \psi(x,x_{0}) \bigr\Vert ^{m}. $$

### Remark 2.4

It is clear that efficient solution implies quasi-efficient solution of order *m* with respect to *ψ* to the (NMP), but the converse may not be true. To illustrate this fact, we consider the following multi-objective programming problem:

$$ \textstyle\begin{cases} \mbox{minimize } f(x)=(\ln(x+1)-x^{2},x^{3}-x^{2})^{T}, \\ \quad \mbox{subject to } x\geq0, \end{cases} $$

where $f: \mathbb{R}_{+}\rightarrow\mathbb{R}^{2}$. Then $x_{0}=0$ is a quasi-efficient solution of order *m* for (NMP) with respect to $\psi (x,x_{0})=(x,x_{0})^{T}$ for $\alpha=(1,1)^{T}$ and $m=2$, but not an efficient solution.

### Remark 2.5

A quasi-efficient solution of order *m* for (NMP) with respect to *ψ* is not to be a quasi-efficient solution in the sense of Definition 2.11. For example, let $X=\{x\in\mathbb{R}:0\leq x\leq1\}$ and $f:X\rightarrow\mathbb{R}^{2}$ be defined as $f(x)=(-x^{4},-\sin^{4} x)^{T}$, then $x_{0}=0$ is not a quasi-efficient solution in the sense of Definition 2.11, because, for any $\alpha=(\alpha_{1},\alpha_{2})^{T}\in \operatorname{int}(\mathbb{R}^{2}_{+})$, there exists an *x* satisfying $x\geq\alpha _{1}^{\frac{1}{3}}$, $\frac{\sin^{4}x}{x}\geq\alpha_{2}$ such that $f(x)\leqslant f(x_{0})-\alpha\|x-x_{0}\|$; however, $x_{0}=0$ is a quasi-efficient solution of order $m=4$ for (NMP) with respect to $\psi (x,x_{0})=(x-\sin^{4}x_{0},x_{0})^{T}$ for $\alpha=(1,1)^{T}$.

Associated with the problem (NMP), we consider the following generalized (weakly) vector variational-like inequalities problems:

(GVVI): Find a point $x_{0}\in X$ such that there exists no $x\in X$ such that
  $$ A\eta(x,x_{0})\leqslant0, \quad \forall A\in\partial_{J} f(x_{0}). $$
(GWVVI): Find a point $x_{0}\in X$ such that there exists no $x\in X$ such that
  $$ A\eta(x,x_{0})< 0, \quad \forall A\in\partial_{J} f(x_{0}). $$

## Relations between (GVVI), (GWVVI) and (NMP)

In this section, by using the tools of nonsmooth analysis, we shall disclose that the solutions of generalized vector variational-like inequalities (GVVI) or (GWVVI) are the generalized quasi-efficient solutions under the extended invexity (defined in Section 2).

### Theorem 3.1

*Let* $f: X\rightarrow\mathbb{R}^{p}$ *be approximately invex of order* *m* *at* $x_{0}\in X$ *with respect to* *η* *and* *ψ*. *If* $x_{0}$ *solves* (GVVI), *then* $x_{0}$ *is a quasi*-*efficient solution of order* *m* *for* (NMP) *with respect to the same* *ψ*.

### Proof

Suppose that $x_{0}$ is not a quasi-efficient solution of order *m* for (NMP) with respect to *ψ*, then there exist $\hat{x}\in X$ and $\alpha\in \operatorname{int}(\mathbb{R}^{p}_{+})$ such that

3.1 $$ f(\hat{x})\leqslant f(x_{0})-\alpha \bigl\Vert \psi( \hat{x},x_{0}) \bigr\Vert ^{m}. $$

Since *f* is approximately invex of order *m* at $x_{0}$ with respect to *η* and *ψ* on *X*, it follows from Definition 2.6 that

3.2 $$ f(\hat{x})\geqq f(x_{0})+A\eta(\hat{x},x_{0})- \alpha \bigl\Vert \psi(\hat {x},x_{0}) \bigr\Vert ^{m}, \quad \forall A\in\partial_{J} f(x_{0}). $$

From inequalities (3.1) and (3.2), we see that there exists $\hat{x}\in X$ such that

$$ A\eta(\hat{x},x_{0})\leqslant0, \quad \forall A\in \partial_{J} f(x_{0}), $$

which is inconsistent with the fact that $x_{0}$ solves (GVVI). □

### Theorem 3.2

*Let* $f: X \rightarrow\mathbb{R}^{p}$ *be approximately invex of order* *m* *at* $x_{0}\in X$ *with respect to* *η* *and* *ψ*. *If* $x_{0}$ *solves* (GWVVI), *then* $x_{0}$ *is a weakly quasi*-*efficient solution of order* *m* *for* (NMP) *with respect to the same* *ψ*.

### Proof

Assume that $x_{0}$ is not a weakly quasi-efficient solution of order *m* for (NMP) with respect to *ψ*, then there exist $\alpha\in \operatorname{int}(\mathbb{R}^{p}_{+})$ and $\hat{x}\in X$ such that

$$ f(\hat{x})< f(x_{0})-\alpha \bigl\Vert \psi(\hat{x},x_{0}) \bigr\Vert ^{m}. $$

Because *f* is approximately invex of order *m* at $x_{0}$ with respect to *η* and *ψ* on *X*, therefore, in particular for $\alpha\in \operatorname{int}(\mathbb{R}^{p}_{+})$ and *x̂*, we have

$$ f(\hat{x})\geqq f(x_{0})+A\eta(\hat{x},x_{0})-\alpha \bigl\Vert \psi(\hat {x},x_{0}) \bigr\Vert ^{m}, \quad \forall A\in\partial_{J} f(x_{0}). $$

Furthermore, we arrive at

$$ A\eta(\hat{x},x_{0})< 0, \quad \forall A\in\partial_{J} f(x_{0}), $$

which contradicts the hypothesis that $x_{0}$ solves (GWVVI). □

### Theorem 3.3

*Let* $f: X \rightarrow\mathbb{R}^{p}$ *be approximately pseudoinvex type I of order* *m* *at* $x_{0}\in X$ *with respect to* *η* *and* *ψ*. *If* $x_{0}$ *solves* (GWVVI), *then* $x_{0}$ *is a weakly quasi*-*efficient solution of order* *m* *for* (NMP) *with respect to the same* *ψ*.

### Proof

Suppose $x_{0}$ solves (GWVVI) but is not a weakly quasi-efficient solution of order *m* for (NMP) with respect to *ψ*, then there exist $\alpha\in \operatorname{int}(\mathbb{R}^{p}_{+})$ and $\hat{x}\in X$, satisfying

3.3 $$ f(\hat{x})< f(x_{0})-\alpha \bigl\Vert \psi(x,x_{0}) \bigr\Vert ^{m}. $$

Noticing that *f* is approximately pseudoinvex type I of order *m* at $x_{0}$ with respect to *η* and *ψ* on *X*, it follows from Definition 2.8 and inequality (3.3) that there exist $\alpha\in \operatorname{int}(\mathbb{R}^{p}_{+})$ and *x̂* such that

3.4 $$ A\eta(\hat{x},x_{0})< 0, \quad \forall A\in \partial_{J} f(x_{0}). $$

This is obviously not in agreement with the hypothesis that $x_{0}$ solves (GWVVI). □

## Characterization of generalized quasi-efficient solutions by vector critical points

This section is devoted to investigating the relations between vector critical points and weakly quasi-efficient solution of order *m* for (NMP) with respect to *ψ* under generalized invexity (introduced in Section 2) hypotheses imposed on the involved functions.

### Definition 4.1

see [4]

A feasible solution $x_{0}\in X$ is said to be a vector critical point of (NMP), if there exists a vetor $\xi\in\mathbb{R}^{p}$ with $\xi\geqslant0$ such that $\xi^{T} A=0$ for some $A\in\partial_{J} f(x_{0})$.

### Lemma 4.1

see [23] (Gordan’s theorem)

*Let* *A* *be a* $p\times n$ *matrix*. *Then exactly one of the following two systems has a solution*:

1. $Ax<0$ *for some* $x\in\mathbb{R}^{n}$.
2. $A^{T}y=0$, $y\geqslant0$ *for some nonzero* $y\in\mathbb{R}^{p}$.

### Theorem 4.1

*Let* $x_{0}\in X$ *be a vector critical point of* (NMP) *and* $f: X\rightarrow\mathbb{R}^{p}$ *be approximately pseudoinvex type I of order* *m* *at* $x_{0}$ *with respect to* *η* *and* *ψ*, *then* $x_{0}$ *is a weakly quasi*-*efficient solution of order* *m* *for* (NMP) *with respect to* *ψ*.

### Proof

Let $x_{0}$ be a vector critical point of (NMP), it follows from Definition 4.1 that there exist a vector $\xi\in\mathbb{R}^{p}$ with $\xi\geqslant0$ and $A\in\partial_{J}f(x_{0})$ such that

$$ \xi^{T} A=0. $$

By contradiction, suppose that $x_{0}$ is not a weakly quasi-efficient solution of order *m* for (NMP) with respect to *ψ*, then for any $\alpha\in \operatorname{int}(\mathbb{R}^{p}_{+})$ there exists an $\hat{x}\in X$ satisfying

4.1 $$ f(\hat{x})< f(x_{0})-\alpha \bigl\Vert \psi( \hat{x},x_{0}) \bigr\Vert ^{m}. $$

Noticing that *f* is approximately pseudoinvex type I of order *m* at $x_{0}$ with respect to *η* and *ψ* on *X*, it follows from Definition 2.8 and inequality (4.1) that

$$ A\eta(\hat{x},x_{0})< 0,\quad \forall A\in\partial_{J}f(x_{0}). $$

Using Gordan’s theorem, the system

$$\begin{aligned}& \xi^{T} A=0, \quad \forall A\in\partial_{J}f(x_{0}), \\& \xi\geqslant0, \quad \xi\in\mathbb{R}^{p}, \end{aligned}$$

has no solution for *ξ*, which contradicts the fact that $x_{0}$ is a vector critical point of (NMP). □

### Theorem 4.2

*Any vector critical point is a weakly quasi*-*efficient solution of order* *m* *for* (NMP) *with respect to* *ψ*, *if and only if* $f: X\rightarrow \mathbb{R}^{p}$ *is approximately pseudoinvex type I of order* *m* *at that point with respect to* *η* *and* *ψ*.

### Proof

The sufficient condition is obtained by Theorem 4.1. In the following we only need to prove the necessary condition. Let $x_{0}$ be a weakly quasi-efficient solution of order *m* for (NMP) with respect to *ψ*, then there exists $\alpha\in \operatorname{int}(\mathbb {R}^{p}_{+})$ such that, for any $x\in X$, the following cannot hold:

4.2 $$ f(x)-f(x_{0})< -\alpha \bigl\Vert \psi(x,x_{0}) \bigr\Vert ^{m}. $$

Noticing that $x_{0}$ is a vector critical point, then there exist $\xi \in\mathbb{R}^{p}$ with $\xi\geqslant0$ and $A\in\partial_{J} f(x_{0})$ such that

$$ \xi^{T} A=0. $$

Using Gordan’s theorem, there exists $A\in\partial_{J}f(x_{0})$ such that the system

$$ \mu^{T} A< 0 $$

has no solution $\mu\in\mathbb{R}^{p}$. Thus, the system

4.3 $$ \mu^{T} A< 0, \quad \forall A\in\partial_{J}f(x_{0}), $$

has no solution $\mu\in\mathbb{R}^{p}$. Therefore, (4.2) and (4.3) are equivalent. Hence, if $x_{0}$ is a weakly quasi-efficient solution of order *m* for (NMP) with respect to *ψ*, that is, for any $\alpha\in \operatorname{int}(\mathbb{R}^{p}_{+})$, there exists no $x\in X$ such that

$$ f(x)-f(x_{0})< -\alpha \bigl\Vert \psi(x,x_{0}) \bigr\Vert ^{m}, $$

then $x_{0}$ solves (GWVVI), that is, there exists no $x\in X$ with $\eta(x,x_{0})\in\mathbb{R}^{p}$ satisfying

$$ A\eta(x,x_{0})< 0, \quad \forall A\in\partial_{J}f(x_{0}). $$

This satisfies the condition of the approximately pseudoinvexity of type I of order *m* of *f* at $x_{0}$. □

## Conclusions

In the current work, we present several extended approximately invex vector-valued functions of higher order involving a generalized Jacobian. Furthermore, the notions of higher-order (weak) quasi-efficiency with respect to a function for a multi-objective programming are also introduced, and some examples are given to illustrate their existence. Under generalization of higher-order approximate invexities assumptions, it proves that the solutions of generalized vector variational-like inequalities in terms of the generalized Jacobian are the generalized quasi-efficient solutions to nonsmooth multi-objective programming problems (*i.e.* Theorems 3.1-3.3). In addition, we also focused on examining the equivalent conditions. By employing the Gordan theorem [23], the equivalent conditions are obtained, that is, a vector critical point is a weakly quasi-efficient solution of higher order with respect to a function (Theorem 4.1 and Theorem 4.2).
